# American College of Sports Medicine (ACSM) International Multidisciplinary Roundtable report on physical activity and nonalcoholic fatty liver disease

**DOI:** 10.1097/HC9.0000000000000108

**Published:** 2023-03-30

**Authors:** Jonathan G. Stine, Michelle T. Long, Kathleen E. Corey, Robert E. Sallis, Alina M. Allen, Matthew J. Armstrong, David E. Conroy, Daniel J. Cuthbertson, Andres Duarte-Rojo, Kate Hallsworth, Ingrid J. Hickman, Matthew R. Kappus, Shelley E. Keating, Christopher J.A. Pugh, Yaron Rotman, Tracey G. Simon, Eduardo Vilar-Gomez, Vincent Wai-Sun Wong, Kathryn H. Schmitz

**Affiliations:** 1 Division of Gastroenterology and Hepatology, Department of Medicine, The Pennsylvania State University—Milton S. Hershey Medical Center, Hershey, Pennsylvania, USA; 2 Department of Public Health Sciences, The Pennsylvania State University—College of Medicine, Hershey, Pennsylvania, USA; 3 Section of Gastroenterology, Evans Department of Medicine, Boston University School of Medicine, Boston, Massachusetts, USA; 4 Division of Gastroenterology and Hepatology, Department of Medicine, Massachusetts General Hospital, Boston, Massachusetts, USA; 5 Department of Family Medicine and Sports Medicine, Kaiser Permanente Medical Center, Fontana, California, USA; 6 Division of Gastroenterology and Hepatology, Department of Medicine, Mayo Clinic, Rochester, Minnesota, USA; 7 Liver Transplant Unit, Queen Elizabeth University Hospitals Birmingham and NIHR Birmingham BRC, Birmingham, UK; 8 Department of Kinesiology, The Pennsylvania State University, University Park, Pennsylvania, USA; 9 Department of Cardiovascular and Metabolic Medicine, Institute of Life Course and Medical Sciences, University of Liverpool, Liverpool, UK; 10 Department of Medicine, Division of Gastroenterology and Hepatology, Northwestern University, Chicago, Illinois, USA; 11 Newcastle NIHR Biomedical Research Centre and the Liver Unit, Newcastle Upon Tyne Hospitals NHS Foundation Trust, Newcastle Upon Tyne, UK; 12 Department of Nutrition and Dietetics, Princess Alexandra Hospital, Brisbane, Queensland, Australia; 13 Division of Gastroenterology and Hepatology, Duke University, Durham, North Carolina, USA; 14 School of Human Movement and Nutrition Sciences, The University of Queensland, St Lucia, Queensland, Australia; 15 Cardiff School of Sport & Health Sciences, Cardiff Metropolitan University, Cardiff, UK; 16 Liver & Energy Metabolism Section, Liver Diseases Branch, National Institute of Diabetes and Digestive and Kidney Diseases, National Institutes of Health, Bethesda, Maryland, USA; 17 Division of Gastroenterology and Hepatology, Indiana University School of Medicine, Indianapolis, Indiana, USA; 18 Department of Medicine and Therapeutics, The Chinese University of Hong Kong, Hong Kong, Hong Kong

## Abstract

We present findings from the inaugural American College of Sports Medicine (ACSM) International Multidisciplinary Roundtable, which was convened to evaluate the evidence for physical activity as a means of preventing or modifying the course of NAFLD.

**Approach and Results:**

A scoping review was conducted to map the scientific literature and identify key concepts, research gaps, and evidence available to inform clinical practice, policymaking, and research. The scientific evidence demonstrated regular physical activity is associated with decreased risk of NAFLD development. Low physical activity is associated with a greater risk for disease progression and extrahepatic cancer. During routine health care visits, all patients with NAFLD should be screened for and counseled about physical activity benefits, including reduction in liver fat and improvement in body composition, fitness, and quality of life. While most physical activity benefits occur without clinically significant weight loss, evidence remains limited regarding the association between physical activity and liver fibrosis. At least 150 min/wk of moderate or 75 min/wk of vigorous-intensity physical activity are recommended for all patients with NAFLD. If a formal exercise training program is prescribed, aerobic exercise with the addition of resistance training is preferred.

**Conclusions:**

The panel found consistent and compelling evidence that regular physical activity plays an important role in preventing NAFLD and improving intermediate clinical outcomes. Health care, fitness, and public health professionals are strongly encouraged to disseminate the information in this report. Future research should prioritize determining optimal strategies for promoting physical activity among individuals at risk and in those already diagnosed with NAFLD.

## Graphical abstract

**Figure:**
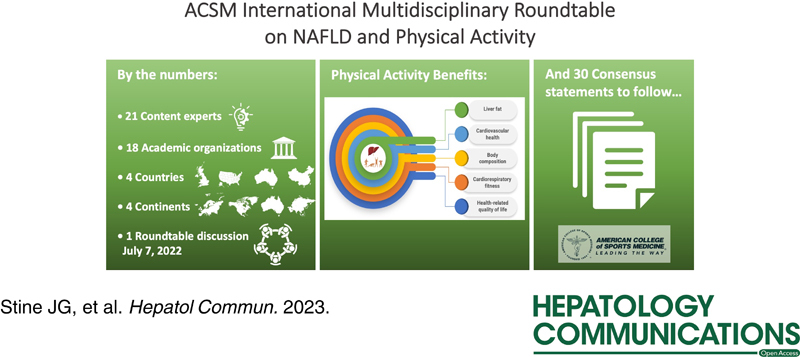

## INTRODUCTION

NAFLD remains a leading cause of morbidity and mortality globally with 25% of individuals having this condition worldwide.^1^–^3^ To date, there is no regulatory agency–approved effective drug therapy or cure, and lifestyle modification with dietary changes and increased physical activity remains the foundation of NAFLD treatment. However, most patients with NAFLD do not meet recommended amounts of weekly physical activity, and the consequences of end-stage liver disease, including liver transplantation and HCC, continue to grow.^4^,^5^

In 2012, several leading gastroenterology and hepatology societies, including the American Association for the Study of Liver Diseases (AASLD), the American Gastroenterology Association (AGA), and the American College of Gastroenterology (ACG) released the initial clinical practice guideline for patients with NAFLD.^6^ In this joint document, the importance of physical activity and, in particular, exercise (defined as a subtype of physical activity that is planned, structured, and repetitive and has the goal of improvement or maintenance of physical fitness), began to be recognized.^6^ Following this, additional guidance from the European Association for the Study of the Liver (EASL) and independent guidance from AASLD were released in 2016 and 2018 respectively with even greater attention to leading a healthy lifestyle;^7^,^8^ yet, these guidelines remained focused on lifestyle intervention as a vehicle for weight loss rather than focusing on the potential weight-neutral benefits of regular exercise. Consequently, rates of physical activity counseling and referral to exercise specialists for tailored exercise prescription remain low in clinical practice.^9^,^10^

To further encourage physical activity in patients with NAFLD, the American College of Sports Medicine (ACSM) and their Exercise is Medicine (EIM) initiative was expanded to include NAFLD in 2018.^11^ However, the EIM recommendations were based largely on expert opinion. Since the original EIM guidance, there has been a substantial accumulation of new evidence supporting the role of physical activity as a means of preventing or modifying the course of NAFLD.

For these reasons, the ACSM convened the inaugural International Multidisciplinary Roundtable on NAFLD and physical activity in July 2022 with the objectives of reviewing and summarizing the biologic, epidemiological, and interventional evidence for the role of physical activity in patients with NAFLD. The purpose of this manuscript is to present the Roundtable findings and conclusions that focused on the relationship between physical activity and NAFLD in terms of (1) NAFLD pathogenesis; (2) screening, advising, and counseling patients with NAFLD about physical activity; (3) providing physical activity recommendations to patients with NAFLD, and (4) outlining future research directions.

## METHODS

The ACSM International Multidisciplinary Roundtable on NAFLD was held virtually (in part due to COVID-19 restrictions) on July 7, 2022, with 21 representatives from 18 organizations globally who were invited to participate based on their clinical and research expertise. Three broad topic areas were addressed, including (1) the role of physical activity in NAFLD pathogenesis; (2) screening, advising, and counseling adult patients with NAFLD about physical activity; and (3) providing physical activity recommendations to adult patients with NAFLD. Following individual scoping reviews of the assigned topics (Supplementary Materials, http://links.lww.com/HC9/A219), each presenter provided a 20-minute overview of their topic followed by a direct question and answer session with the entire Roundtable Faculty. Herein, we provide an overview of the Roundtable presentations and discussions.

## PATHOGENESIS OF NAFLD AND MECHANISMS EXPLAINING THE BENEFIT OF PHYSICAL ACTIVITY

**FIGURE 1:**
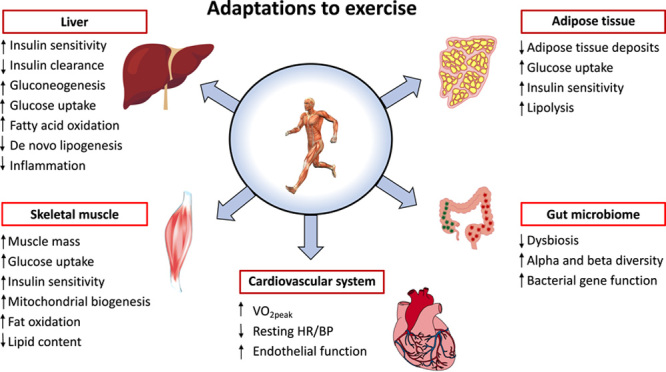
Adaptations to exercise training in patients with NAFLD. Abbreviations: BP, blood pressure; HR, heart rate; VO_2_peak, peak oxygen uptake.

Despite decades of research, NAFLD remains a complex, progressive disease process with many different pathogenic mechanisms and inputs.^12^ Regular physical activity, in particular moderate-intensity aerobic exercise training,^13^ can impact several pathogenic factors and may also influence gene expression.^14^,^15^ While the mechanisms by which physical activity benefits patients with NAFLD remain poorly understood and understudied in patients,^16^,^17^ regular physical activity, including exercise training, can lead to significant adaptations in not only the liver, but also in skeletal muscle and adipose tissue, each of which communicate with the liver through myokines and adipocytokines (Figure 1), and also the cardiovascular system.^16^,^18^–^22^

Exercise training also impacts the microbiome and gut-liver-axis dysfunction.^23^ Patients with NAFLD have significant dysbiosis with an overabundance of Gram-negative bacteria, which can lead to gut barrier dysfunction and a “leaky gut.”^24^,^25^ After as little as 20 weeks of moderate-intensity aerobic exercise training, many beneficial effects have been observed on the gut-liver-axis in patients with NAFLD, including reversal of dysbiosis with the restoration of healthy bacterial balance as well as improvements in alpha (species richness) and beta diversity (species diversity).^13^,^23^ Moreover, aerobic exercise training impacts bacterial gene expression in a way that may remove many of the pathogenic factors implicated in the development of NAFLD (Figure 1).^13^,^23^

## EPIDEMIOLOGICAL EVIDENCE: ASSOCIATION BETWEEN PHYSICAL ACTIVITY AND NAFLD

To date, there is a plethora of epidemiological data supporting an association between NAFLD and physical activity, including multiple large cross-sectional studies using nationwide databases.^26^–^32^ Collectively, these studies have found a consistent body of evidence that meeting or exceeding guideline-based physical activity amounts is associated with a decreased risk of incident NAFLD by roughly 50%. Perhaps more importantly, over 10+ years of follow-up, patients with NAFLD who perform regular moderate-to-vigorous physical activity have lower overall and cardiovascular disease (CVD) mortality.^27^ Similar trends for incident NAFLD reduction hold true when physical activity is completed in small, continuous bouts of aerobic activity of at least 10 minutes in length,^33^–^35^ provided the overall amount of physical activity completed is still the same. Unfortunately, despite this large body of epidemiological evidence, most patients with NAFLD do not meet current physical activity guidelines and spend more time pursuing sedentary behaviors.^10^,^36^,^37^ Specifically, patients with NAFLD walk, on average, ∼10 km less each week than those without NAFLD, and each additional hour of sedentary behavior is associated with 1.2% higher liver steatosis content.^37^,^38^

While the relationship between physical activity and risk of hepatic or extrahepatic malignancy in patients with NAFLD remains less explored, indirect evidence can be extrapolated from general population-based studies which show regular physical activity is associated with a lower risk of multiple primary cancers, including both HCC and nonhepatic cancers, which are found in greater rates in patients with NAFLD.^39^–^41^ Based on these data, it is possible to hypothesize that in NAFLD, regular physical activity could decrease the risk of developing HCC and several other extrahepatic cancers, however, at this time, there is no robust evidence directly linking those factors together and future studies are needed to confirm this.

## BENEFITS OF PHYSICAL ACTIVITY

**FIGURE 2:**
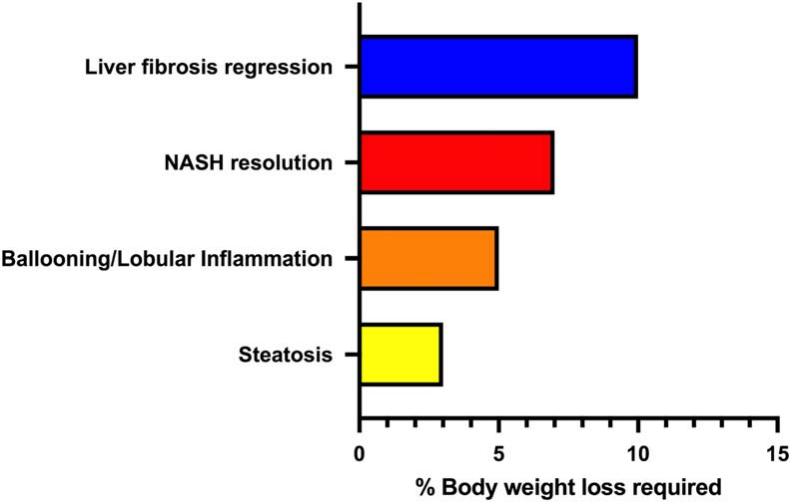
Expected change in liver histology in patients with NASH across different thresholds of body weight loss.

There are many well-established benefits of regular physical activity and, in particular, exercise training in patients with NAFLD and NASH, as detailed below.^17^ Importantly, some of these benefits may be independent of clinically significant weight loss,^42^,^43^ including improvements in liver fat (as determined by MRI), cardiovascular health, body composition, cardiorespiratory fitness, and health-related quality of life (HRQOL). Whether weight loss is required for histologic improvement with exercise training is controversial, however, in general, it is accepted that various thresholds of weight loss would be expected to lead to specific histologic changes in patients with NASH (Figure 2).^44^–^46^

### Reduction in liver fat content

The most widely studied and established benefit of exercise training in patients with NAFLD is an improvement in MRI-measured liver fat. Upwards of 25 studies have documented that exercise training can improve liver fat in adults with NAFLD, the majority of which appear to be independent of clinically significant weight loss.^20^,^47^–^61^ Importantly, these studies utilize many different types of exercise training programs, including aerobic training, resistance training, high-intensity interval training (HIIT), and aerobic and resistance training combined. Beyond this, individual studies have reported rates of liver fat reduction similar to that seen in early-phase NASH drug trials^43^ and at a threshold of response (a relative reduction of >30%) that may surrogate for corresponding histologic improvement in NASH activity and liver fibrosis stage.^62^,^63^

### Histologic improvement (NAFLD activity score)

To date, 4 clinical trials have demonstrated exercise training alone, or in combination with dietary modification, can favorably influence liver histology in patients with NAFLD and NASH; however, these studies are limited by significant heterogeneity and sample size with <60 patients being studied in total.^64^–^67^ Following 3 months of aerobic exercise training, Hickman et al^65^ demonstrated steatosis and liver fibrosis stage improvement. Eckard et al^66^ measured change in liver histology following 6 months of moderate-intensity aerobic exercise and found no statistically significant improvement in NAFLD activity score. O’Gorman et al^64^ observed improvement in hepatocyte ballooning (67%) and liver fibrosis stage (58%) following 12 weeks of aerobic exercise training; however, no changes were seen in overall NAFLD activity score or 2 of its individual components in steatosis or lobular inflammation, raising questions about the clinical significance of the histologic change.^68^ Promrat et al^67^ combined 200 min/wk of unsupervised moderate-intensity aerobic exercise with a hypocaloric diet over 40 weeks and found nearly 75% of patients had NAS reduction or NASH resolution. Each of these studies that reported statistically significant changes in liver histology also observed concomitant reductions in body weight loss.^64^,^65^,^67^ Accordingly, it is currently unclear whether exercise training can independently improve liver histology in the absence of weight loss.

### Cardiovascular health

CVD is a leading cause of death in patients with NAFLD and for this reason it remains a focus of tremendous research and clinical interest.^69^ The body of research about cardiovascular health and exercise in patients with NAFLD and NASH relates to the improvement of CVD biomarkers including improvements in endothelial dysfunction and changes in serum plasminogen activator inhibitor-1 concentration. Endothelial dysfunction leads to abnormal blood flow and the development of arterial plaque that over time can rupture and lead to arterial thrombosis.^70^,^71^ Independent of traditional CVD risk factors, endothelial dysfunction represents the earliest manifestation of atherosclerosis and is found globally in NAFLD.^72^–^76^ Green et al^77^ found a 16-week moderate-intensity aerobic exercise protocol reversed endothelial dysfunction suggesting this intervention may have benefit in the primary prevention of coronary artery disease in patients with NAFLD. Unfortunately, in their follow-up study,^74^ in patients who were no longer exercising 12 months after the intervention concluded, the improvement in endothelial dysfunction was no longer evident. In addition, the NASHFit study^43^ found a notable reduction in plasminogen activator inhibitor-1 compared with standard clinical care after 20 weeks of moderate-intensity aerobic exercise.

### Change in body composition: adipose tissue volume and lean body mass

Multiple studies have demonstrated visceral adipose tissue is reduced with aerobic exercise training.^43^,^73^,^78^–^82^ Fewer studies explored the impact of exercise training on subcutaneous adipose tissue, however, the recent NASHFit study^43^ found a significant reduction in subcutaneous adipose tissue after 20 weeks of aerobic exercise training. Importantly, this was much more common in patients who also met the clinically significant threshold of relative reduction in MRI-measured liver fat.^62^,^63^ To date, no individual study has found a significant change in lean body mass with an exercise intervention, including those studies that used resistance training programs, albeit these were of short duration.^43^,^83^–^87^

### Improvements in cardiorespiratory fitness

Patients with NAFLD have lower cardiorespiratory fitness (maximal oxygen uptake; VO_2_peak) than the general population.^36^,^88^ The majority of patients with NAFLD have a poor or very poor fitness level, independent of traditional metabolic risk factors and other hallmark predictors of fitness such as age, body weight, and sex.^89^ Importantly, cardiorespiratory fitness is predictive of mortality^90^–^93^ and may be associated with liver disease severity.^36^,^94^ It is widely accepted that exercise training improves cardiorespiratory fitness with multiple meta-analyses reporting a pooled increase in maximal oxygen uptake^48^,^60^,^83^ at the clinically significant threshold required to improve overall mortality.^92^ In addition, various exercise training modalities have all led to improvement in cardiorespiratory fitness including moderate-intensity aerobic exercise and HIIT.^43^,^48^,^57^,^60^,^83^

### Improvement in HRQOL

Patients with NAFLD have low HRQOL in comparison to the general population^95^ and other types of chronic liver disease.^96^ While exercise training is known to improve HRQOL in the general population and in individuals with chronic disease, including diabetes,^97^,^98^ routine assessment of this has not traditionally been included in the design and conduct of exercise-based interventional trials outside of a recent study which demonstrated exercise training to lessen pain interference and strengthen social roles.^43^

**FIGURE 3:**
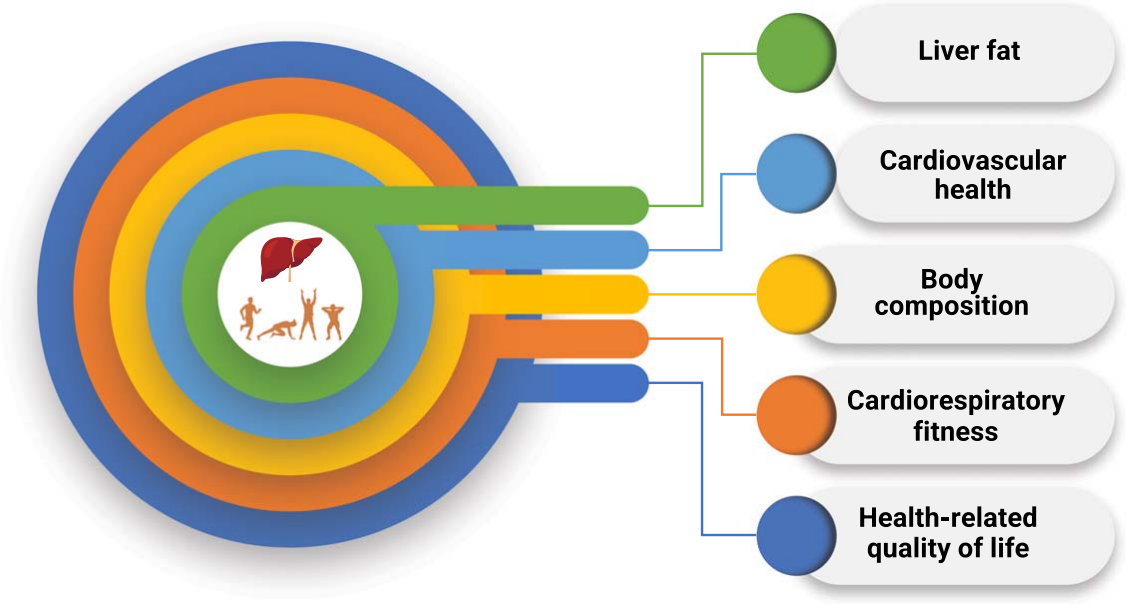
Expected benefits of physical activity in patients with NAFLD.

Figure 3 summarizes each of the expected benefits of physical activity in patients with NAFLD.

## ASSESSMENT AND SCREENING FOR PHYSICAL ACTIVITY

Several research-based tools have been validated for the routine assessment of physical activity in the general population. These tools include the Get Active Questionnaire,^99^ International Physical Activity Questionnaire,^100^ and Physical Activity Readiness Questionnaire.^101^ Because these tools can be time-consuming in clinical practice, the ACSM developed the 2 question Physical Activity Vital Sign,^102^,^103^ (https://exerciseismedicine.org/wp-content/uploads/2021/04/EIM-Physical-Activity-Vital-Sign.pdf) which calculates the volume of moderate-to-vigorous physical activity completed (Table 1). Importantly, the Physical Activity Vital Sign can be readily integrated into the electronic health record and if elected, can assess the number of days per week spent performing muscle-strengthening exercises. Whether these tools are cost-effective at the population level or lead to direct improvement in clinical outcomes in patients with NAFLD remains unknown.

**TABLE 1:** American College of Sports Medicine (ACSM) Physical Activity Vital Sign^102^,^103^ (https://exerciseismedicine.org/wp-content/uploads/2021/04/EIM-Physical-Activity-Vital-Sign.pdf)

1. On average, how many days per week do you engage in moderate to vigorous physical activity (like a brisk walk)?	—— days
2. On average, how many minutes do you engage in physical activity at this level?	—— minutes
Total minutes per week of physical activity (multiple #1 by #2)	—— minutes per week

Screening for barriers preventing physical activity should also be routinely performed in patients with NAFLD. While no one optimal tool has been identified, the Screening, Brief Intervention, and Referral to Treatment (SBIRT) may be considered.^104^ In the general population, the Centers for Disease Control and Prevention (CDC) has identified 8 common barriers to physical activity including a lack of time, social support, energy, motivation, or skill and also high cost, fear of injury, and poor weather conditions.^105^ Patients with NAFLD have similar self-reported barriers, although a lack of knowledge about exercise prescription in health care providers was also identified as a barrier to physical activity.^10^ Key unanswered questions remain about physical activity barrier assessment including what the optimal interval of assessment is and how health care providers can tackle the barriers effectively. In addition, data on the impact of assessment on clinical outcomes are lacking.

## COUNSELING ABOUT PHYSICAL ACTIVITY

When counseling patients about physical activity, it is first important to understand what factors (barriers or enablers) determine whether a person engages or not in behavior. A patient’s capabilities, opportunities, and motivation all play a role.^106^ An understanding within and across different levels of social-ecological influence is necessary to identify which of those determinants are related to the change in behavior. When considering specific behavior change techniques and interventions for patients with NAFLD, there is a dearth of scientific literature. What exists focuses largely on validating exercise as a behavioral target for improving patient health through evidence that supervised exercise training improves fitness and NAFLD-related biomarkers of treatment response.^107^

When considering a recommendation for health care providers to counsel patients on exercise, there are no efficacy or comparative effectiveness trials of physician counseling about the benefits of physical activity among patients with NAFLD. However, in non-NAFLD populations with metabolic disease, physician counseling, including the importance of engaging multidisciplinary care teams to promote physical activity has been studied.^108^ Nevertheless, few studies have looked at the efficacy of these efforts perhaps because counseling and education alone are rarely enough to enact long-term lifestyle change. The use of patient-centered language should be encouraged within all counseling sessions. Stigmatizing language about body weight and body image should be avoided.^109^ Unfortunately, nearly three-fourth of patients with NAFLD reported experiencing stereotypical judgments, discrimination, shame, and social isolation at one point in time,^110^ which can lead to poor HRQOL and a reduction in help-seeking behavior.

Several techniques have been used to promote behavior change in patients with NAFLD. Motivational interviewing, which is a technique that is feasible to utilize within a 20-minute clinic visit, can empower patients with NAFLD to make individual health-related decisions,^111^ however, the majority of health care providers are not trained in this technique.^112^,^113^ Despite this lack of widespread training, motivational interviewing has been used in patients with NAFLD as a way to reduce body weight through dietary change and increased physical activity.^114^ Cognitive behavioral therapy is an additional technique that has also been used successfully in patients with NAFLD to increase physical activity, with gains in fitness and loss of body weight that can persist for up to 2 years.^115^ Lifestyle intervention based on social-cognitive theory, which explains how an individual’s motivation derives from the reciprocal interactions between a person, their behavior, and their environment that shape a person’s beliefs and goals, has also been applied to promote physical activity in patients with NAFLD.^116^,^117^ Print and web-based patient education materials have been successful modes for promoting health behavior change in patients without NAFLD,^118^ however, in patients with NAFLD, including those from the ACSM EIM initiative, there is a lack of evidence about the effectiveness of providing these materials. Critically, these modes are just vehicles for delivering intervention content. The degree to which that content exposes patients to evidence-based behavior change techniques will account for specific mode effects on behavior change.

Patients with advanced liver disease, including those with cirrhosis, represent a unique patient population that needs to be viewed independently of patients with earlier stage disease. A recent systematic review summarized the 11 small clinical trials limited exclusively to patients with cirrhosis, including 4 studies which used home-based interventions,^119^ and suggests exercise training to be feasible and safe in patients with cirrhosis, including those whose primary etiology of liver disease is NASH.^120^–^123^ In terms of efficacy, individual studies have shown regular exercise training can decrease portal hypertension,^120^,^124^ and may also improve physical performance, frailty, and HRQOL.^121^,^122^,^125^–^127^ However, these studies did not enroll patients with decompensated cirrhosis and the feasibility, safety, and efficacy of exercise training in this patient population remains unknown and we look to future and ongoing research to help answer this key question.

## REFERRING A PATIENT WITH NAFLD TO AN EXERCISE SPECIALIST

Because physicians are not routinely trained in exercise prescription during their medical education, we propose that the exercise specialist should be a key member of the NAFLD treatment team. Avery et al^128^ performed structured interviews with health care professionals and patients with NAFLD and found the majority of physicians felt ill-equipped to address lifestyle behavior change with their patients with NAFLD, choosing to monitor rather than actively manage dietary change and physical activity. Patients felt a multidisciplinary team including an exercise specialist, rather than an individual physician, would offer personalized support with the goal of achieving long-term behavior change.

Once a patient with NAFLD is referred to an exercise specialist, a key focus of the assessment should be to evaluate the patient’s understanding of what NAFLD is and how it relates to their lifestyle behaviors. Additional factors to consider include (1) the patient’s goals for treatment; (2) measurement of current and historical levels of physical activity; (3) assessment of cardiometabolic risk factors, comorbidities, past medical history, and current medications; (4) evaluation of baseline levels of cardiorespiratory fitness as well as functional and exercise capacity; and (5) identification of barriers and facilitators to physical activity participation.

To assist the exercise specialist, the ACSM has established guidelines to determine when a medical referral is recommended before starting an exercise training program.^129^ These guidelines rely on the following: current exercise participation; history and symptoms of cardiovascular, metabolic, or renal disease; and the desired exercise intensity for the person who wants to initiate a physical activity program. Because the vast majority of patients with NAFLD do not exercise regularly and have a history of either metabolic or CVD, medical guidance from their treating physician may be required before starting an exercise program.

When the exercise specialist is designing a training program for patients with NAFLD, the most important consideration is how to tailor exercise for each individual, taking into account baseline capabilities, comorbidities, and personal preferences. Once an exercise program has commenced, there are many management priorities the exercise specialist should be aware of. These include targeting improvement in body weight/body composition, glycemic control, CVD risk factors (eg, blood pressure), cardiorespiratory fitness as well as functional and exercise capacity. The exercise specialist can also play a key role in counseling patients with NAFLD on alcohol intake, smoking cessation, and sleep quality and duration and may be a central person to initiate a referral to other members of the multidisciplinary care team including a dietitian or psychologist where appropriate.

## CONSIDERATIONS FOR PRESCRIBING AN EXERCISE TRAINING PROGRAM

Exercise training is well-established as a key component in the clinical management of patients with NAFLD. Previous large systematic reviews, including a recent Cochrane review and those with quantitative meta-analysis, have examined the individual components of the ACSM FITT exercise prescription, including frequency, intensity, time, and type.^47^,^57^,^107^,^130^ Each has found significant heterogeneity owing to variation in the FITT principles. Across the primary literature, exercise frequency ranges from 3 to 7 days/wk; intensities that have been studied include low, moderate, moderate-vigorous, and vigorous. Time in each exercise bout is also widely variable and ranges from 20 to 60 minutes. Exercise types which have been studied include aerobic exercise training, resistance training, combined aerobic with resistance training, HIIT, and pilates. Moreover, almost all exercise interventions have been carried out under direct supervision.

Despite decades of research, no single optimal exercise prescription has been defined for patients with NAFLD. While the majority of studies have used aerobic exercise training and head-to-head direct comparisons of different exercise modalities are generally lacking, indirect evidence suggests that resistance training may have a role.^20^ A recent meta-analysis by Hashida et al^20^ found equivalent improvement in clinical outcomes when comparing similar volumes of resistance training to aerobic exercise. Importantly, resistance training required less energy consumption and can be considered in patients who cannot tolerate or participate in aerobic exercise programs. Along the same lines, Sabag et al^57^ performed a meta-analysis and compared HIIT to moderate-intensity aerobic exercise training and found both exercise types to reduce liver fat in similar amounts. The appropriateness of other types of physical activity including yoga and Pilates remains uncertain.^131^,^132^

While most exercise training programs for patients with NAFLD have relied on in-person supervised training, several recent studies have explored the role of telehealth as a means to increase adherence and access to exercise programs. Huber et al^133^ found an 8-week web-based exercise program to improve cardiorespiratory fitness, liver biochemistries, and transient elastography-measured liver fat. Motz et al^134^ used audiovisual telehealth technology to directly supervise a small group of patients with biopsy-proven NASH in a 20-week aerobic exercise training program. No adverse events were observed and clinical benefits exceeded those reported for traditional in-person aerobic exercise or resistance training programs. Other studies have used unsupervised, in-person resistance training^135^ and found that after 3 months, improvements in hepatic fat content were accompanied by favorable changes in body composition.

## FUTURE DIRECTIONS—KEY KNOWLEDGE GAPS AND LIMITATIONS IN THE CURRENT SCIENTIFIC LITERATURE

Following a review of the available primary literature, the ACSM Roundtable identified key knowledge gaps in the existing scientific literature in terms of (1) the role of physical activity in NAFLD pathogenesis; (2) assessing and screening patients with NAFLD for physical activity; (3) advising and counseling patients with NAFLD about the benefits of physical activity; (4) physical activity recommendations in patients with NAFLD; and (5) referring a patient with NAFLD to an exercise specialist (Table 2).

**TABLE 2:** Key knowledge gaps that remain about the relationship between NAFLD and physical activity

*Role of physical activity in NAFLD pathogenesis:*
1. What are the mechanisms underlying the suggested benefit of physical activity in patients with NAFLD?
2. What role does the microbiome play in the association between physical activity and NAFLD?
*Assessing and screening patients with NAFLD for physical activity:*
3. What is the most appropriate setting for physical activity screening and/or intervention, primary care or specialist (eg, hepatologist) clinic?
4. In patients who are physically inactive, should physical function or frailty testing be routinely performed?
5. Should patients with NAFLD be routinely screened for sarcopenia?
*Advising and counseling patients with NAFLD about the benefits of physical activity:*
6. How can rates of lifestyle counseling be increased in the primary care and specialist setting?
7. What is the minimum amount of physical activity at varying intensities that a patient with NAFLD needs to complete in order to achieve clinically meaningful benefit?
8. What is the role of personalized medicine in exercise prescription in patients with NAFLD?
9. Do patients with NAFLD, who are more physically active, experience greater treatment responses when exercise is prescribed in combination with pharmacologic therapy for NAFLD?
*Physical activity recommendations in patients with NAFLD:*
10. What is the most health-enhancing physical activity prescription for patients with NAFLD?
11. Does sustained physical activity directly lead to improvement in long-term outcomes such as cardiovascular disease events, liver and extrahepatic cancer, major adverse liver outcomes, or death?
12. What are predictors (eg, genetic, physiological, psychological) of exercise response in patients with NAFLD?
13. Is home-based/virtually supervised exercise as effective as supervised in-person exercise in patients with NAFLD?
14. Does the addition of improved diet quality lead to greater clinically meaningful benefit of physical activity?
15. Does the addition of pharmacologic obesity treatment as an adjunct to physical activity improve liver-related and non–liver-related outcomes (eg, CVD)?
16. What factors are associated with patients developing motivation for physical activity, initiating an increase in physical activity and sustaining physical activity over time?
*Referring a patient with NAFLD to an exercise specialist:*
17. How can suboptimal rates of referral to exercise specialists be increased?
18. How can we best ascertain which patients would benefit the most from referral to an exercise specialist (eg, all patients versus those with either early stage or advanced liver disease)?
19. Will engaging multiple stakeholders, including patients, lead to greater rates of exercise specialist referral?
20. Will closer multidisciplinary care with an exercise specialist in the same clinical space improve patient-oriented outcomes?

Abbreviation: CVD, cardiovascular disease.

Because liver fibrosis is closely related with long-term outcomes in patients with NASH, histologic improvement remains the goal of all NASH clinical trials.^136^,^137^ At this point in time, it is unclear whether exercise intervention can improve liver histology without clinically significant weight loss. Determining the independent impact of an exercise intervention on liver histology remains a clear, unmet need of high significance and even greater impact and should be prioritized by research funding and public health agencies as a way to answer this important question.

Many of these knowledge gaps and key unanswered questions remain either from a lack of investigation or from limitations inherent to the scientific literature where included populations across all study designs are heterogenous and often ill-defined. In fact, the majority of interventional studies include all stages of NAFLD with only 3 trials limited only to NASH.^43^,^67^,^85^ Moreover, there is a lack of standardization of exercise intervention, where exercise training programs vary significantly in components of FITT exercise prescription or measurement of adherence and clinical outcomes. The behavioral science literature is even more limited where the literature is largely limited to small, observational studies with a high risk of bias.

In light of these limitations, we look to future grant funding mechanisms to support the research of the highest rigor to answer each and every one of the remaining key knowledge gaps (Table 2).

## CONCLUSIONS

An expert panel reviewed the published scientific evidence and came to a consensus regarding the role of physical activity in patients with NAFLD. The evidence supports that regular physical activity is associated with decreased risk of NAFLD development and that low physical activity is associated with a greater risk for disease progression and extrahepatic cancer. Physical activity screening during routine health care visits in all patients with NAFLD would seem prudent. Moreover, this visit offers a unique opportunity to open a discussion about the many benefits of regular physical activity, many of which may be weight loss independent. If weight loss does occur, this can provide additional benefits. Currently, at least 150 min/wk of moderate or 75 min/wk of vigorous-intensity physical activity are recommended for all patients with NAFLD. If a formal exercise program is to be used, the combination of aerobic plus resistance training is preferred, albeit emerging data suggests that HIIT can be considered in select patients instead. Multiple research gaps remain in this field, including the need for studies exploring the mechanistic multiorgan underpinnings of exercise’s benefit, best counseling practices, exercise dosing, and predictors of exercise response. Health care, fitness, and public health professionals are strongly encouraged to disseminate the information in this report and to encourage and support all patients with NAFLD to be as physically active as their age, abilities, and environment will allow. Future research should prioritize determining optimal strategies for promoting physical activity among individuals at risk and in those already diagnosed with NAFLD.

## Supplementary Material
