# Supplementary material for: American College of Sports Medicine (ACSM) International Multidisciplinary Roundtable report on physical activity and nonalcoholic fatty liver disease
Source: Hepatol Commun. 2023 Mar 30;7(4):e0108. doi: 10.1097/HC9.0000000000000108 (PMC10069861; doi:10.1097/HC9.0000000000000108)
Supplement: Supplementary file 1 [file hc9-7-e0108-s001.docx]

**Scientific Committee Members**

Committee Co-Chairs:

Kathryn H. Schmitz PhD, MPH, FACSM, FTOS, Past ACSM President

Jonathan G. Stine MD MSc, FACP; Research Director Penn State Liver Center, ACSM member

Committee Members:

Robert E. Sallis, MD, Exercise is Medicine Advisory Board Chairman, ACSM

Michelle Long, MD MSc, NAFLD Research Director, Boston University Medical Center

Kathleen Corey, MD MPH, Massachusetts General

**Faculty Members**

Alina Allen, MD

Mayo Clinic

Matthew Armstrong, MrCP, PhD

University Hospitals Birmingham NHS Foundation Trust

Dan Cuthbertson, PhD

University of Liverpool

David E. Conroy, PhD

Pennsylvania State University

Andres Duarte-Rojo, MD MS, PhD

Northwestern University

Kate Hallsworth, PhD

Newcastle Biomedical Research Center

Ingrid Hickman, PhD

University of Queensland

Matthew Kappus MD

Duke University

Shelly Keating, PhD, AEP, AES, ESSAM

University of Queensland

Christopher Pugh, PhD

Cardiff Metropolitan University

Mary Rinella, MD

University of Chicago

Yaron Rotman, MD MSc, FAASLD

National Institutes of Health

Tracey L. Simon, MD MPH

Massachusetts General Hospital

Eduardo Vilar-Gomez, MD PhD, MSc

Indiana University

Vincent Wong, MBChB, MD, FRCP, FHKCP, FHKAM

University of Hong Kong

**Roundtable Agenda- Thursday July 7, 2022 10a-7p EST**

**1000-1010a Opening Remarks and History of ACSM Roundtables**

Dr. Jonathan G. Stine, MD MSc, FACP

Dr. Kathryn H. Schmitz PhD MPH, FACSM, FTOS

**1010-1020a ACSM Exercise is Medicine initiative**

Dr. Robert E. Sallis, MD (pre-recorded)

**Session 1 Role of physical activity in NAFLD pathogenesis**

**1020-1040a What is NAFLD?**

Dr. Alina Allen, MD

**1040-1100a NAFLD pathogenesis**

Dr. Yaron Rotman, MD MSc, FAASLD

**1120-1140a NAFLD and physical activity: Epidemiological evidence**

Dr. Tracey Simon, MD MPH

**1140-1200p** **What is the mechanism of exercise’s benefit in patients with NAFLD?**

Dr. Andres Duarte-Rojo, MD MS, PhD

**1200-1220p Group Discussion**

Moderator: Dr. Michelle Long, MD MSc

**1220-120p** **Lunch Break**

**Session 2 Screening, advising and counseling patients with NAFLD about physical activity**

**120-140p** **Assessing and screening patients with NAFLD for physical activity**

Dr. Vincent Wong, MBChB, MD, FRCP, FHKCP, FHKAM

**140-200p How to counsel patients with NAFLD about physical activity**

Dr. David E. Conroy, PhD

**200-220p** **What are the benefits of physical activity for patients with NAFLD?**

Dr. Eduardo Vilar-Gomez, MD PhD, MSc

**220-240p Group Discussion**

Moderator: Dr. Kathleen Corey, MD MPH

**240-300p Break**

**Session 3 Physical activity recommendations in patients with NAFLD**

**300-320p Referring a patient with NAFLD to an exercise specialist**

Dr. Kate Hallsworth PhD (pre-recorded)

**320-340p Exercise prescription for patients with NAFLD**

Dr. Kathryn H. Schmitz, PhD, MPH, FACSM, FTOS, Past ACSM President

**340-400p Group Discussion**

Moderator: Dr. Jonathan G. Stine, MD MSc

**400-420p Break**

**Session 4 Consensus statement and key unmet research needs in using exercise as medicine in NAFLD**

**420-550p Consensus statements (with levels of evidence) on NAFLD exercise prescription**

Group discussion

Moderator: Dr. Jonathan G. Stine, MD MSc

**550-600p Break**

**600-650p Key unmet research needs**

Group discussion

Moderator: Dr. Kathryn H. Schmitz PhD, MPH, FACSM, FTOS

**650-700p Closing remarks**

Dr. Jonathan G. Stine, MD MSc, FACP

Dr. Kathryn H. Schmitz PhD, MPH, FACSM, FTOS

**700p Adjournment**
